# Supplementary material for: Maize Inbred Line B96 Is the Source of Large-Effect Loci for Resistance to Generalist but Not Specialist Spider Mites
Source: Front Plant Sci. 2021 Jun 21;12:693088. doi: 10.3389/fpls.2021.693088 (PMC8256171; doi:10.3389/fpls.2021.693088)
Supplement: Supplementary file 1 [file Data_Sheet_1.PDF]

Supplementary Material

Maize inbred line B96 is the source of large-effect loci for resistance to generalist but not specialist spider mites

Huyen Bui, Robert Greenhalgh, Gunbharpur S. Gill, Meiyuan Ji, Andre H. Kurlovs, Christian Ronnow, Sarah Lee, Ricardo A. Ramirez, and Richard M. Clark*

* Correspondence: Richard M. Clark: richard.m.clark@utah.edu

1 Supplementary Figures

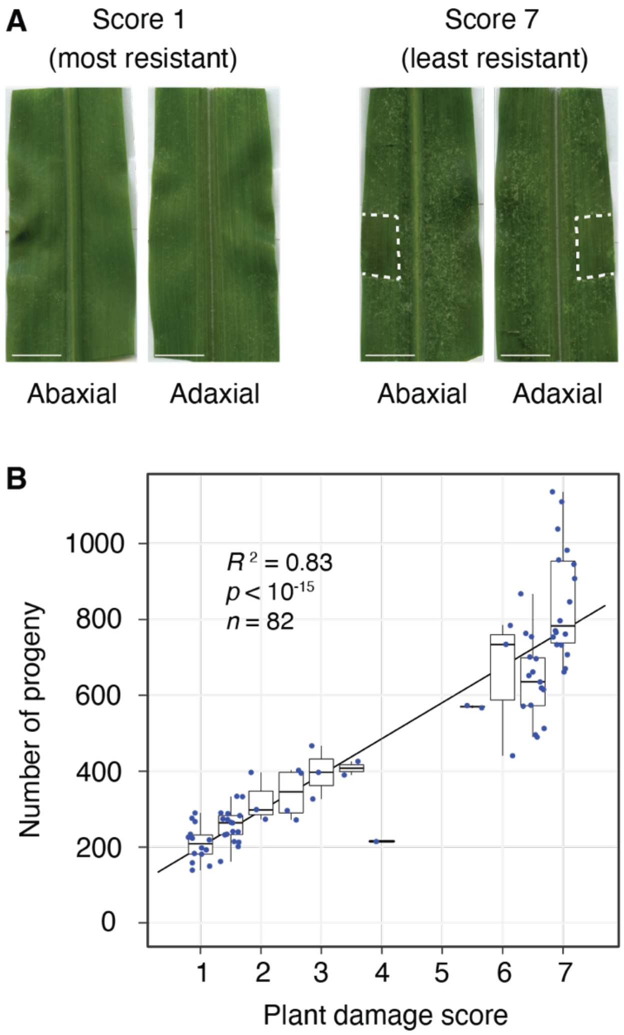

Supplementary Figure 1. Relationship between plant damage and mite reproduction in a cross between the *T. urticae* resistant maize line B49 and susceptible B73. (**A**) An image for a representative highly resistant sample (score 1) and a representative highly susceptible sample (score 7) following six days of *T. urticae* feeding (4th leaves are displayed). Damage to the lower (abaxial) and upper (adaxial) leaf blades are shown; the leaf segments were excised from within Tanglefoot enclosures for image capture. For the resistant sample (right), a region of reduced feeding (outlined by dashed white lines) corresponds to where tape was affixed to apply the pipet tip of mites for release into the leaf blade enclosure (see Materials and Methods). Scale bars: 1 cm. (**B**) Boxplots (plant damage scores, plotted in units of 0.5, with jitter of data points by bin) showing the relationship between the visual plant damage score and mite productivity – as assessed by the number of progeny counted following six days of addition of adult *T. urticae* females to enclosures – for a subset of plants used for BSA genetic mapping (data shown are for replicate two of the B49 cross, Supplementary Figure 2B). Blue circles represent individual data points.

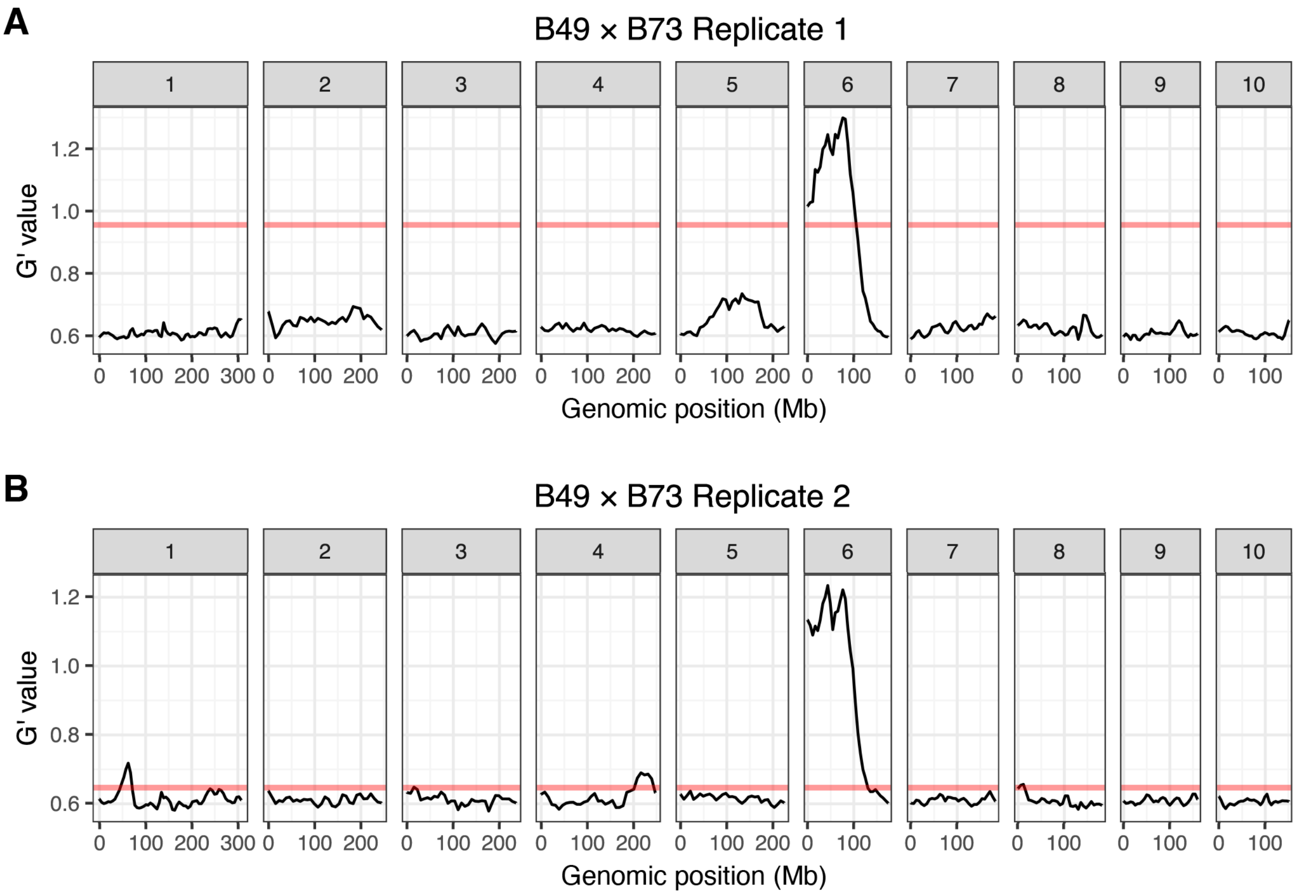

Supplementary Figure 2. BSA genetic mapping scans for QTL for resistance to *T. urticae* strain W-GR in two F2 population replicates of the B49 × B73 cross. The plots shown correspond to that of **Figure 4A**, except that the BSA analysis was done on a per replicate basis as indicated, top and bottom (see the legend for **Figure 4** for additional details). The solid red line denotes a genome-wide FDR of 0.01 for QTL detection.

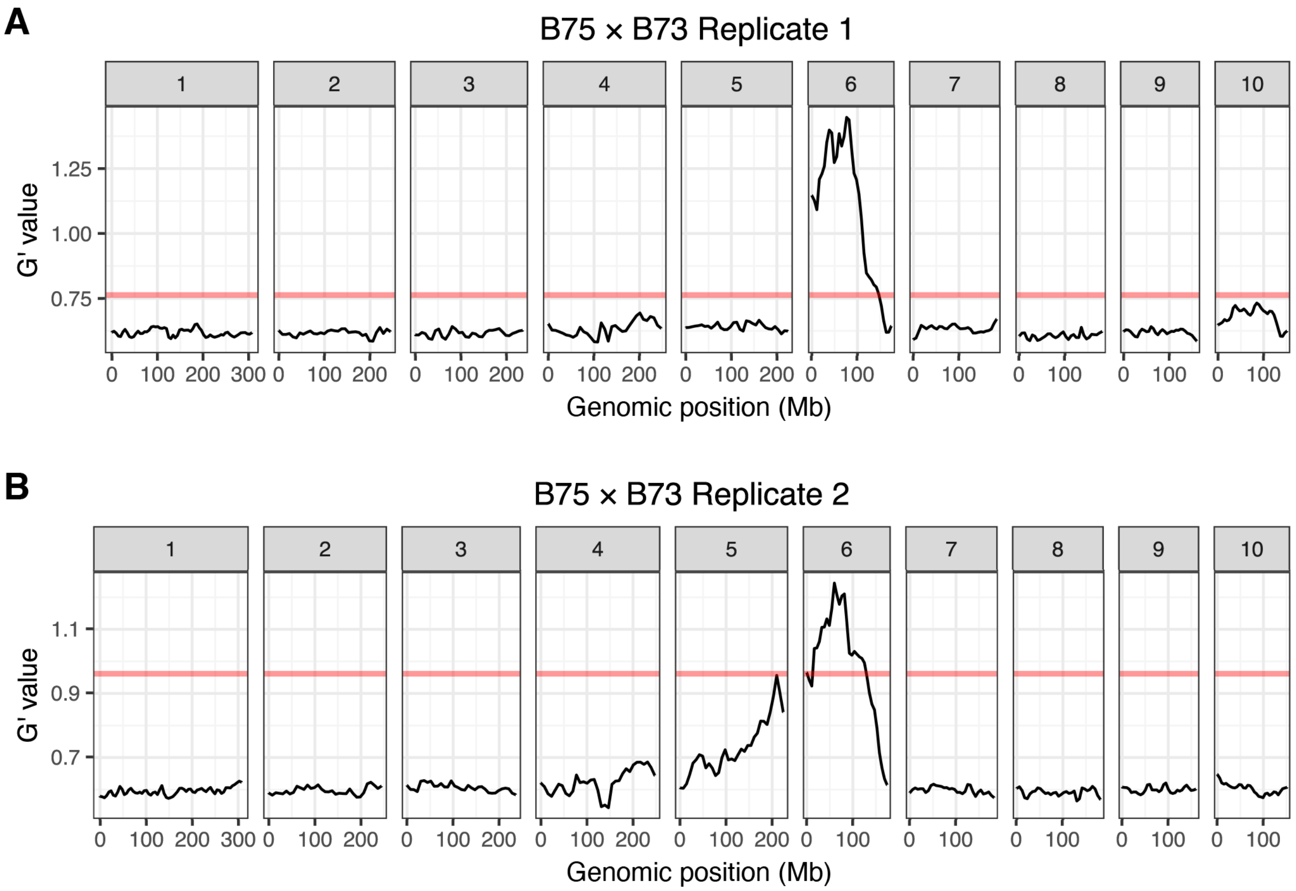

Supplementary Figure 3. BSA genetic mapping scans for QTL for resistance to *T. urticae* strain W-GR in two F2 population replicates of the B75 × B73 cross. The plots shown correspond to that of **Figure 4B**, except that the BSA analysis was done on a per replicate basis as indicated, top and bottom (see the legend for **Figure 4** for additional details). The solid red line denotes a genome-wide FDR of 0.01 for QTL detection.

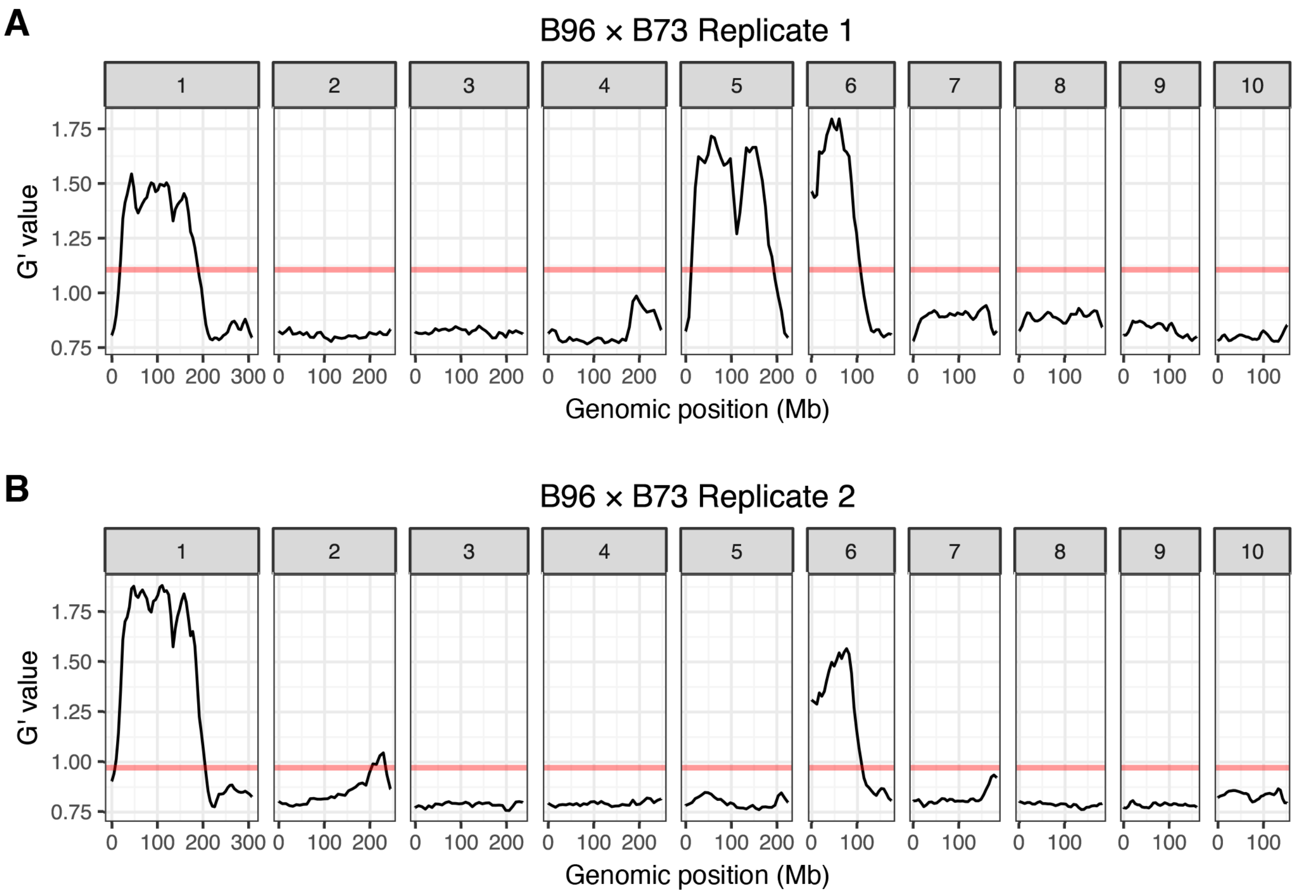

Supplementary Figure 4. BSA genetic mapping scans for QTL for resistance to *T. urticae* strain W-GR in two F2 population replicates of the B96 × B73 cross. The plots shown correspond to that of **Figure 4C**, except that the BSA analysis was done on a per replicate basis as indicated, top and bottom (see the legend for **Figure 4** for additional details). The solid red line denotes a genome-wide FDR of 0.01 for QTL detection.

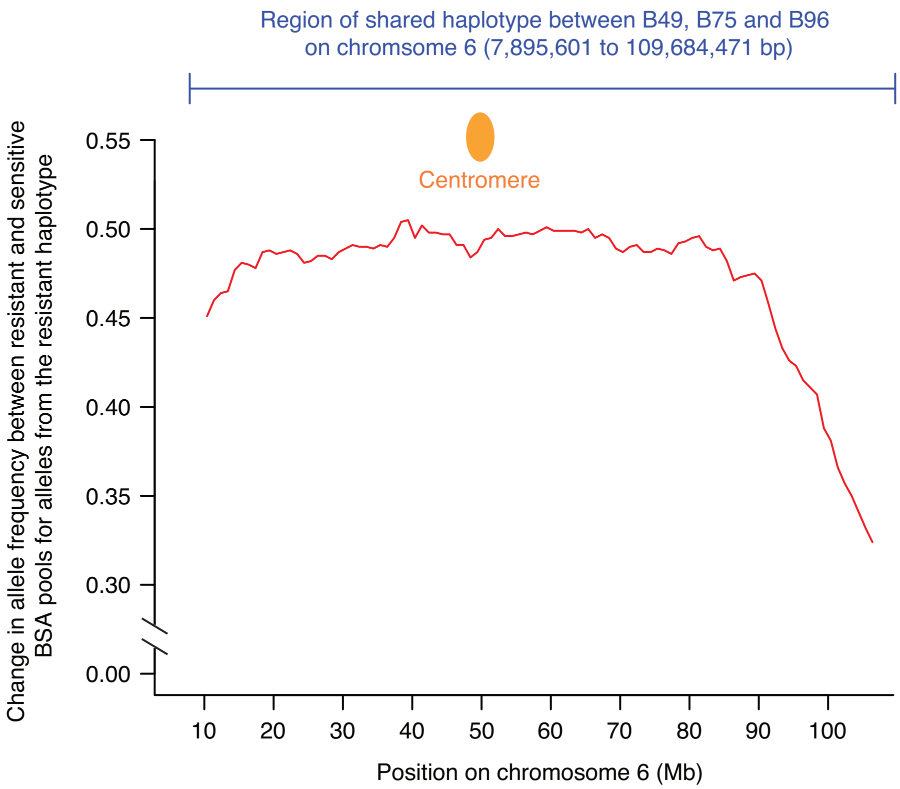

Supplementary Figure 5. Resolution of the genomic interval for a *T. urticae* resistance QTL on chromosome 6. Shown is the difference in the frequency of resistant alleles (those shared between B49, B75, and B96) as compared to the total (resistant and sensitive, with latter from B73) for the region of the shared haplotype between B49, B75, and B96 on chromosome 6 (denoted in blue at top, and see **Figure 6**) as assessed between the combined BSA resistant pool data and the combined sensitive pool data (red line; see Methods). The combined resistant and sensitive pool data were derived by merging all the resistant and sensitive BSA pool data from all replicates of all crosses of resistant lines B49, B75, and B96 to sensitive B73 (Supplementary Figures 2-4). The maximal plateau region from about 31 to 86 Mb defines the apparent location of the QTL, with decreases in the difference of the frequency of the resistant alleles between the resistant and sensitive pools apparent approximately equidistant from the centromere location (indicated at top, orange). For the sliding window analysis, windows of 5 Mb were used with 1 Mb step sizes.
